# Dynamic changes of driver genes’ mutations across clinical stages in nine cancer types

**DOI:** 10.1002/cam4.704

**Published:** 2016-03-19

**Authors:** Xia Li

**Affiliations:** ^1^ Laboratory for Gene and Cell EngineeringShenzhen Institute of Advanced TechnologyChinese Academy of Sciences1068 Xueyuan AvenueShenzhen University TownShenzhenChina

**Keywords:** Hotspot, mutations, TCGA, temporal order, tumorigenesis

## Abstract

The driver genes play critical roles for tumorigenesis, and the number of identified driver genes reached plateau. But how they act during different cancer development stages is lack of knowledge. We investigated 138 driver genes’ mutation changes across clinical stages using 3,477 cases in nine cancer types from the Cancer Genome Atlas (TCGA) and constructed their temporal order relationships. We also examined the codon changes for the widely mutated *TP53* and *PIK3CA* in tumor stages. Combinations of one to three driver genes specifically dominated in each cancer. Across the clinical stages, we categorized three patterns for the behaviors of driver genes’ mutation changes in the nine cancer types: recurrently mutated in all the stages and triggering other mutations; certain mutations lost meanwhile other mutations emerged; mutations dominated across entire stages, while other mutations gradually appeared or disappeared. We observed different codon changes dominated in different stages and revealed mutations recurrently occurring on the hotspot regions of the coding sequence may be the core factor for driver genes’ tumorigenesis. Our results highlighted the dynamic changes of oncogenesis roles in different clinical stages and suggested different diagnostic decision making according to the clinical stages of patients.

## Introduction

Cancer is a disease largely driven by variety of genetic abnormalities. An average of 33–66 genes harbor somatic mutations that result in their protein products changes 1. During the tumorigenesis, each mutation played specific role, but not all mutations were critical to propagate the cancer cells. A mutation that conferred growth advantage and has been positively selected in the microenvironment are called “driver” mutation which is causally implicated in oncogenesis 2. In contrast, a “passenger” mutation which is often found during cell division has no functional contribution on cancer growth 2. Great efforts have been made on the identification of driver genes 3, 4, 5. The newly identified driver genes were also “rediscovered” in other cancer types, suggesting the number of driver genes were nearing saturation 6, 7, 8. One estimation in the lung and colorectal cancers showed that only three driver gene mutations were required for a normal human cell to progress to an advanced cancer 9. Among around 20,000 protein‐coding genes in the human genome, only 138 driver genes were identified in different cancers by previous studies to contribute cancer progression 1. Deciphering the tumorigenesis mechanisms for them is crucial.

The observation that older patients have more mutations and prediction in certain tumors showing the number of mutations correlated with the age of patients indicate the mutations occurred in cancers were matter of timing 1, 10. One best studied example is the temporal order of *APC*,* KRAS*,* PIK3CA*, and *TP53* mutations at the transitions between each tumor stage in colorectal cancer 11, 12. The first driver mutation provided a selective growth advantage to normal cells. With clinical stage increasing, the appearance of a second driver mutation unleashed a second round of growth. These four driver genes mutated separately in different time, finally driving the cancer cells developing from early stage to advanced stage. Targeting the key driver mutations according to the tumor stage may make it efficient to inhibit the tumor growth. However, it is unclear whether the driver gene mutations in other cancer type showed any patterns during the tumorigenesis stages.

The large volume of cancer data in the Cancer Genome Atlas (TCGA) now provide one rich resource and opportunity to comprehensively investigate the mutations across many cancer types. Here, we used total 3,477 cases’ of exome‐seq data across nine cancer types from TCGA to analyze the 138 public‐reported driver genes’ mutation changes in different stages. We observed the behaviors of these genes in the nine cancer types can be categorized into three patterns based on their dynamic mutation changes during tumor progression. Additionally, we detected different leadership for codon changes in different stages. Interestingly, we found the recurrent codon changes occurring at those hotspot regions of the coding sequence might be critical for the driver gene's tumorigenesis. The analysis highlighted the dynamic changes of oncogenesis roles in different clinical stages, providing some clues for driver genes performance mechanisms and anticancer‐targeted therapies.

## Materials and Methods

### Data

The processed exome‐seq mutation data and clinical information for all cancer types in this study were downloaded from TCGA (TCGA “Level 3” data designation). We filtered those variants located in the 1000 Genome Project 13, the NHLBI GO Exome Sequencing Project 14 which represented more than 200,000 individuals’ variants, and the Exome Aggregation Consortium 15 which spanned 60,706 unrelated individuals’ variants by applying a minor allele frequency threshold of 0.1. Only the nonsynonymous mutations were remained.

The 138 driver genes were obtained from previous report 1. In general, a total of 18,306 mutated genes harboring 404,863 subtle mutations from the Catalogue of Somatic Mutations in Cancer (COSMIC) database were assessed as driver genes and checked whether they were likely to be oncogenes or tumor suppressor genes. The oncogene was classified if >20% of the recorded mutations in the gene occurred at recurrent positions and were missense. If >20% of the recorded mutations in the gene were inactivating, the gene was categorized as a tumor suppressor gene. This “20/20 rule” was applied for the selection of the 138 driver genes and categorizing as oncogenes or tumor suppressor genes (Table S1). We also scanned the literatures for these 138 genes and listed one example of reported cancer type in which the corresponding gene served as driver gene role.

### Data processing

Variants were annotated for effects on transcripts using the variant effector predictor tool 16. The mutation spectrum for the driver genes in each cancer types were extracted according to the mutation status. The patient proportion of each driver genes was calculated based on the number of patients harboring the corresponding driver gene, divided by the total number of patients in each cancer type.

All the patients in each cancer type were divided into subgroups according to their clinical stage information. We discarded those patients if the clinical stage is not available or vague. In each stage subgroup, we calculated the number of patients harboring the driver genes and divided by the total number of patients belonging to that stage subgroup. Similar processing was applied on the 140 randomly selected genes in each cancer type.

For the codon changes, we extracted the amino acids information from the results of variant effector predictor. We calculated the number of patients harboring that specific codon change in each stage subgroup.

### Temporal order inference

In each cancer type, we merged all the driver genes’ mutation into one matrix of genes versus tumor samples with 0/1 entries indicating the absence/presence status of a mutation in a gene for each sample. Based on this matrix, we used the BML 17 tool to infer the temporal order relationship. The low‐probability (probability below 0.4) events were filtered.

### Statistical analysis

The correlation of mutation numbers with patient stages was performed with the R Version 3.1.1 statistical software (R Core Team, Vienna, Austria). *P* value was calculated by two‐sided Student's *t* test, with a significance level of *P *<* *0.01.

## Results

### Patient and association of mutations with clinical stages

We collected a total of 3,477 cases’ exome‐seq data from nine cancer types that represent a wide range of tissues and carcinogenic mechanisms: bladder urothelial carcinoma (BLCA), breast invasive carcinoma (BRCA), cervical squamous cell carcinoma and endocervical adenocarcinoma (CESC), esophageal carcinoma (ESCA), kidney renal clear cell carcinoma (KIRC), liver hepatocellular carcinoma (LIHC), lung adenocarcinoma (LUAD), pancreatic adenocarcinoma (PAAD), and stomach adenocarcinoma (STAD) (Table 1). There were total 894 cases in Stage I, 1,245 cases in Stage II, 815 cases in Stage III, 297 cases in Stage IV, and 12 cases in Stage V (according to the International Federation of Gynecology and Obstetrics [FIGO] staging system 18).

**Table 1:** Cancer types and number of cases from TCGA

Cancer types	Number of cases					
	Total	Stage I^a^	Stage II^a^	Stage III^a^	Stage IV^a^	Stage V^a^
Bladder urothelial carcinoma (BLCA)	404	2	128	139	133	/
Breast invasive carcinoma (BRCA)	987	164	558	222	15	12
Cervical squamous cell carcinoma and endocervical adenocarcinoma (CESC)	198	121	36	30	7	/
Esophageal carcinoma (ESCA)	183	17	81	57	15	/
Kidney renal clear cell carcinoma (KIRC)	491	197	40	112	68	/
Liver hepatocellular carcinoma (LIHC)	202	78	47	58	5	/
Lung adenocarcinoma (LUAD)	560	265	116	82	25	/
Pancreatic adenocarcinoma (PAAD)	163	13	138	5	5	/
Stomach adenocarcinoma (STAD)	289	37	101	110	24	/

a According to the International Federation of Gynecology and Obstetrics (FIGO) staging system. The “/” means no case available for the cancer type.

We observed that the number of nonsynonymous mutations had no correlation with the clinical stages in all cancer types (Fig. S1). This is similar with the observation in another study on Natural killer/T‐cell lymphoma 19. The development of early stage to later stage represents the size growth of the tumor and the metastasis ability acquirement. Our results suggested that some mutations might be lost during the tumor development.

### Combinations of one to three driver genes specifically dominated in each cancer

**Figure 1:**
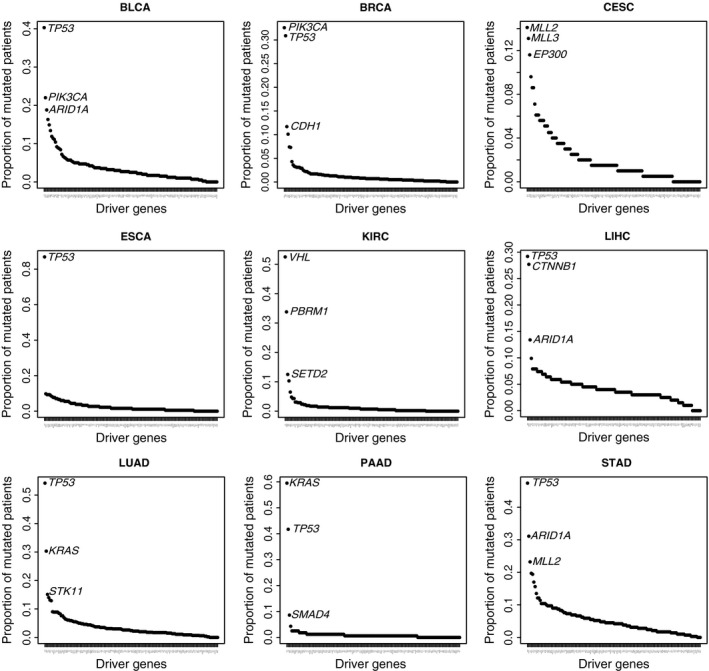
Distribution of patients proportion for the driver genes in nine cancer types.

Given the driver mutations’ important roles for cancer progression, we selected those 138 public reported driver genes for study. The same driver gene mutated in one cancer type may also mutate in other cancer type and also serve as the driver role in that cancer 6, 7, 8. We then looked at the mutated patients’ proportion for each of the 138 driver genes in each cancer. We observed the driver genes generally showed one cancer type specific distribution pattern, with different combinations (Fig. 1). In each cancer, the majority of driver genes did not show high‐mutated patient frequency, while only one to three driver genes were frequently mutated. Among these recurrently mutated driver genes, the mutations on *TP53* widely mutated. While in CESC and KIRC, both of these two cancer types had their own high‐frequency mutations, such as *MLL3*,* EP300*, and *VHL*.

### Three patterns for driver genes’ dynamic mutation changes across clinical stages

**Figure 2:**
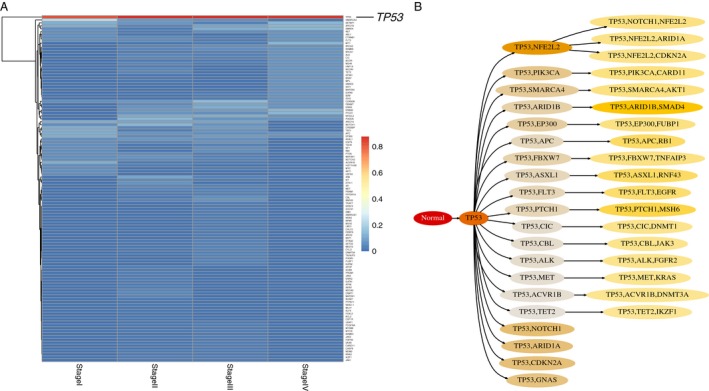
Representative example for the “dominant” pattern. (A) Heat map shows the proportion of patients for driver genes in each clinical stage in esophageal carcinoma (ESCA). We labeled *TP53* which recurrently mutated in different stages. (B) The temporal order relationship of driver genes in ESCA. Figure shows all possible mutation paths for driver genes after leaving out low‐probability (the probability from the parent circle to the child circle below 0.4) events. Color for each circle is scaled according to the relative probability (decreasing from darker shade to light) from the parent circle to the child circle.

**Figure 3:**
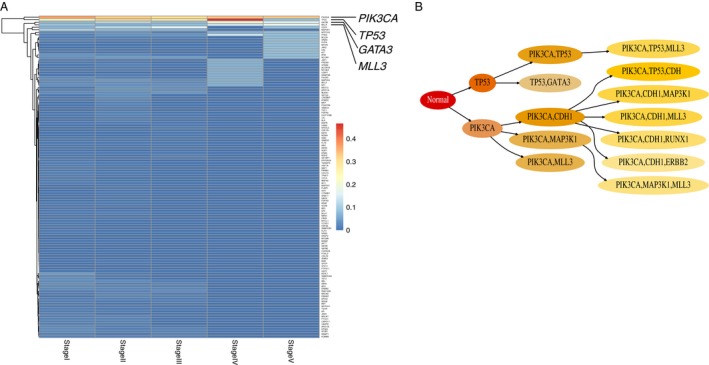
Representative example for the “dominant‐waves” pattern. (A) Heat map shows the proportion of patients for driver genes in each clinical stage in breast invasive carcinoma (BRCA). We labeled the recurrently mutated genes in different stages. (B) The temporal order relationship of driver genes in BRCA. Figure shows all possible mutation paths for driver genes after leaving out low‐probability (the probability from the parent circle to the child circle below 0.4) events. Color for each circle is scaled according to the relative probability (decreasing from darker shade to light) from the parent circle to the child circle.

**Figure 4:**
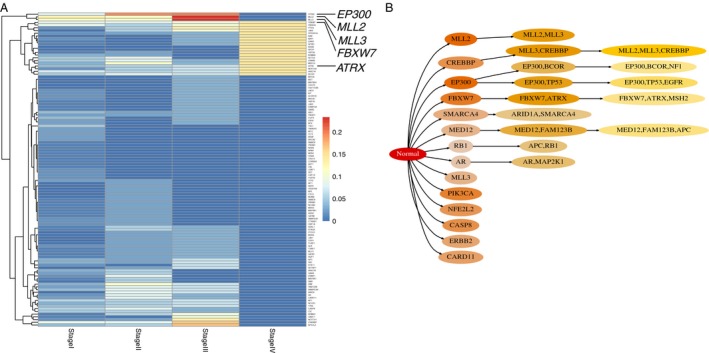
Representative example for the “waves” pattern. (A) Heat map shows the proportion of patients for driver genes in each clinical stage in cervical squamous cell carcinoma and endocervical adenocarcinoma (CESC). (B) The temporal order relationship of driver genes in CESC. Figure shows all possible mutation paths for driver genes after leaving out low‐probability (the probability from the parent circle to the child circle below 0.4) events. Color for each circle is scaled according to the relative probability (decreasing from darker shade to light) from the parent circle to the child circle.

We divided the patients into subgroups according to their clinical stages and calculated the patients proportion for each driver gene in each stage subgroup. Interestingly, we observed that driver genes mutated differently across the stages. Some mutations recurrently occurred in all stages (Fig. 2A). Some mutations mostly mutated in early stage while disappeared in later stage, and some showed opposite pattern (Fig. 3A, Fig. 4A). We also randomly selected 140 mutated genes from each cancer type and checked how the non‐driver genes, or mainly passenger genes, are distributed across different stages. Similar dynamic mutation patterns were also observed in those random genes and it seemed more obvious than in driver genes (Fig. S2).

There seemed existing three major patterns for the driver genes’ dynamic mutations. One pattern was obviously observed in ESCA where only *TP53* recurrently mutated in all stages (Fig. 2A). We constructed the temporal order for these driver genes. Consistently, *TP53* played one critical initiation event, other gene mutations like *NFE2L2* were consequent events (Fig. 2B). We named this pattern “dominant” meaning there were mutations predominantly occurring among patients across all stages and triggering other mutations. Similarly, in BLCA and STAD, *TP53* also dominated in all stages (Figs. S3 and S4). In KIRC, both *VHL* and *PBRM1* seemed be the dominant mutations, and *BAP1* and *SETD2* were the following events. In the case of LUAD, although *TP53* occurred in most of the patients in each stage, *KRAS* dominated and triggered other mutations like *STK11* and *ATM*.

In BRCA, another interesting pattern was observed that mutations on *PIK3CA* dominated while other mutations greatly changed (Fig. 3). At the Stage IV ([any T, any N, M1]: the cancer can be any size (any T) and may or may not have spread to nearby lymph nodes (any N). It has spread to distant organs or to lymph nodes far from the breast (M1), *TP53* mutated mostly in patients (patient proportion: 46.7%), even surpassing *PIK3CA* (patient proportion: 33.3%). However, another two gene mutations, *GATA3* and *MLL3* (20% and 13.3% of patients separately), appeared rightly at this stage. When arriving at the last stage, surprisingly, *TP53* almost showed no mutations in patients (patient proportion: 8.3%), while *PIK3CA* still occurred (patient proportion: 33.3%), but *GATA3* and *MLL3* (both 16.7% of patients) were still observed at this stage (Fig. 3A). Combining with the temporal order (Fig. 3B), the dynamic order in this case should be: *PIK3CA* first triggered *TP53*, and *TP53* triggered *GATA3* and *MLL3*, then *TP53* gradually disappeared, but *PIK3CA* still existed. This pattern was more like one dominant mutation together with one wave of mutation following another wave and we named “dominant waves.” Similar pattern was also observed in PAAD (Fig. S5A, Fig. S5C). *KRAS* led the initial and predominant role across all stages. *TP53* generally showed two waves of mutations, separated by Stage III ([T4, Any N, M0]: the tumor is growing outside the pancreas into nearby major blood vessels or nerves (T4). It may or may not have spread to nearby lymph nodes (Any N). It has not spread to distant sites (M0).

The final pattern was best characterized in CESC (Fig. 4), in which HPV infection became one critical factor for this disease. From Stage I ([T1, N0, M0]: the cancer has grown into (invaded) the cervix, but it is not growing outside the uterus. The cancer has not spread to nearby lymph nodes (N0) or distant sites (M0) to Stage III ([T3, N0, M0)]: the cancer has spread to the lower part of the vagina or the walls of the pelvis. The cancer may be blocking the ureters (tubes that carry urine from the kidneys to the bladder). It has not spread to nearby lymph nodes (N0) or distant sites (M0), mutations on *EP300*,* MLL2*, and *MLL3* were predominant, while after Stage III these genes faded away (patient proportion: 0), but other genes such as *FBXW7*,* PIK3CA*,* ERBB2*, and *ATRX* gradually appeared and played one leading role. This pattern followed one wave of mutation and then another wave and we called “waves.” LIHC also showed similar pattern as CESC (Fig. S5B, S5D). *TP53* triggered *CTNNB1*, and then *CTNNB1* triggered another event like *ARID1A*. In the final stage, other genes like *AXIN1*,* BAP1*,* ARID2*,* TSC1*,* NRAS*,* EGFR*,* JAK1*,* RET*, and *SKP2* occurred in more patients. It is unclear whether these genes were consequently followed by *CTNNB1* or *ARID1A* or even events followed by *ARID1A*. But it is obvious that this cancer type also showed two waves of mutations. Interestingly, HBV infection was the greatest risk factor for the LIHC carcinogenesis. The virus infection may be associated with the “waves” pattern of mutations across different clinical stages.

### Different codon changes dominated in different stages

**Figure 5:**
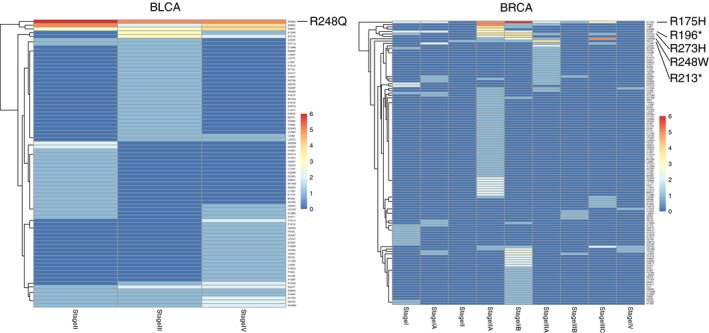
Distribution of codon changes of *TP53* in two cancer types. Heat map shows the number of patients harboring each codon changes of *TP53* in each stage. We labeled the mutations occurred on the hotspot regions.

We noticed *TP53* widely mutated in seven cancer types, either as the “dominant” roles in some cancer types (BLCA, ESCA, STAD) or “waves” in other cancer (BRCA, LIHC, PAAD), or even none of them (LUAD). To determine more details about the specific mutations for this gene, we focused on the amino acids changes in these seven cancer types. We calculated the number of patients harboring each amino acids changes of *TP53* in each stage subgroup and checked the changes across stages. Similar as the observations for driver genes’ mutations described above, only several codon changes recurrently occurred in patients and the codon changes also showed certain dynamics across stages (Fig. 5, Fig. S6). Some codon changes highly occurred in all stages, such as in BLCA the R248Q mutation. While in some cancer types like BRCA, R175H mutated mostly among patients in Stage IIA ([T2 N0 M0]: tumor more than 2 cm but not more than 5 cm in greatest dimension [T2], no regional lymph node metastasis [N0], no distant metastasis [M0]), and Stage IIB ([T3, N0, M0]: tumor is larger than 5 cm across but does not grow into the chest wall or skin and has not spread to lymph nodes (T3, N0). The cancer has not spread to distant sites (M0), but R248W took one leading role in Stage IIIC ([any T, N3, M0]: tumor is any size and has spread to 10 or more axillary lymph nodes (N3), the cancer has not spread to distant sites (M0). Similarly, in LUAD, R280T and E286* in Stage IB ([T2a, N0, M0]: the main tumor is larger than 3 cm across but not larger than 5 cm, has not spread to lymph nodes or distant sites), and D281Y in Stage IIB ([T3, N0, M0]: the main tumor is larger than 7 cm across, and has not spread to lymph nodes or distant sites (Fig. S6).

### Recurrent mutations occurred on the protein hotspot may be critical for tumorigenesis

For *TP53*, there existed certain hotspots, like position of 175, 248, 273, 196, 213, 245, 282, and 306 in the coding sequence 20. All the seven cancer types harbored mutations on these hotspots (Fig. 5, Fig. S6). Only in LIHC, none of these positions was recurrently occurred among patients (R249S mutation occurred mostly) (Fig. S6). This may somehow explain why the mutation occurred in *TP53* in LIHC could not persist in the most advanced stage where *CTNNB1* triggered another waves of mutations (Fig. S5B, Fig. S5D). We noticed earlier that in LUAD, *TP53* mostly mutated among patients while did not contribute much for cancer development (Fig. S3, Fig. S4). Carefully looking at the codon changes distribution (Fig. S6), only three codon changes mostly occurred among patients: R280T, E286*, and D281Y, still these were not the hotspots. This indicated that the mutations occurred at the hotspots might drive the cancer toward one more aggressive stage. In other five cancer types, mutations in these hotspots all occurred mostly among the patients in the corresponding stages. Moreover, *TP53* was able to trigger other mutation events, either in the “dominant” or “wave” role. It seemed only mutations happened in those hotspot regions that can be selected to be the driver. Mutations occurred on other regions may also be recurrent, but they did not contribute much for cancer progression.

Another widely mutated gene was *PIK3CA*, “dominant” role in BRCA and “wave” in CESC. Three hotspots were also identified 21, 22: H1047R in the kinase domain, E542K and E545K in the helical domain. Interestingly, we observed H1047R obviously occurred in Stage IIA ([T2a, N0, M0]: the cancer has not spread into the tissues next to the cervix (called the parametria). The cancer may have grown into the upper part of the vagina. It has not spread to nearby lymph nodes (N0) or distant sites (M0), followed by E545K mutation in BRCA (Fig. S7), where *PIK3CA* served as the “dominant” role (Fig. 3). However, in CESC, none of these three hotspots were observed (Fig. S7). *PIK3CA*, with the “wave” role, none of downstream events appeared with high probability (Fig. 4). Again, these also suggested that the hotspot may serve as critical role for driver gene's tumorigeneisis. It should be noted that in BRCA both *PIK3CA* and *TP53* recurrently mutated among patients, yet only *PIK3CA* dominated in all stages (Fig. 3). It is not clear whether there existed functional competition or cooperation between two or more driver genes, if they at the same time all mutated in one cancer type.

## Discussion

The clinical staging is one important parameter to describe the extent to which the cancer have developed, and also critical for treatment consideration. Driver genes serve as the most important role in tumorigenesis. Determining how their genetic alterations behaved during the tumorigenesis can provide useful diagnostic and therapeutic purposes. In this study, we demonstrated three dynamic patterns for 138 driver genes’ mutation behaviors across different clinical stages in the nine cancer types. Additionally, our results of the codon changes of *TP53* and *PIK3CA* revealed different leaderships for codon changes in different stages and indicated that recurrent mutations on hotspot regions of the driver genes coding sequence may be the core factor for tumorigenesis. These findings expanded our understanding of the mutations of driver genes and especially their dynamic roles during the progression of cancer that would greatly improve therapeutic strategies for cancer.

Cancer results from genetic alterations and the mutational landscape is complex. The selective pressure makes some mutations dominant and sweeps other mutations resulting extinction 23. Previous mathematical model simulated one “travelling wave” growth and extinction pattern of the cancer cell numbers by sequential acquisition of driver mutations 24, 25. The author suggested that it might be due to the competition between the new driver gene and the previous one. This theoretically simulated the genetic instability in tumor progression, but it is unclear whether this pattern of cancer cell growth is in one certain period of time like in certain stage or across all the development stages. Our observations on the nine cancer types also revealed similar wave dynamic pattern which appeared during different development stages. Besides this wave pattern, we observed that the “dominant” and “dominant waves” also existed in some cancer types.

Additionally, we noticed only one to three driver genes occurred mostly in each cancer type, either in certain stages or across the entire stages. The three patterns observed also showed there were only one to three driver genes showed “dominant” or “wave” role in certain stages or across all stages. This is consistent with that previously reported in the lung and colorectal cancers that three driver genes are required for tumor progression 9. This phenomenon was observed in the nine cancer types which might be universal to other cancer types which we currently did not assess. Interestingly, in the BRCA, two driver genes *TP53* and *PIK3CA* both displayed tumorigenesis properties, but *PIK3CA* seemed more aggressive than *TP53*. This is similar as reported previously 26 and there may exist competition between two driver genes 25. Maybe this can also explain why cancer does not need too many driver genes at the same time. More details about the competition or cooperation mechanisms between driver genes are worthy of further study.

Previous cancer studies investigated recurrent mutations in cancer and associated them with the function to promote cancer progression 27. Take the cervical cancer for example, the somatic mutations in *PIK3CA*,* PTEN*,* TP53*,* KRAS*,* EP300*,* FBXW7*,* NFE2L2*, and so on, were recurrently occurred and have been implicated in the pathogenesis of cervical carcinomas 27. Our data agreed with these important potential driver genes, but we put weight on the *EP300*,* MLL2*, and *MLL3* for their predominant carcinogenesis roles before Stage III; but in the late Stage IV we mostly focused on the mutations on other genes such as *FBXW7*,* PIK3CA*,* ERBB2*, and *ATRX*. For the therapy consideration, it suggested that different diagnosis and treatment should be made according to the clinical stages of patients and the boundary for the stage separation can be Stage III.

Interestingly, when we carefully checked the tumor suppressor and oncogene function for the “dominant” or “wave” genes in each cancer type, it is obvious that the tumor suppressor genes mainly mutated in each cancer type. In the “dominant” pattern, there were five cancer types, and all of the genes in the “dominant” roles were tumor suppressor genes, except in LUAD, where *KRAS*, which was an oncogene, dominated in this cancer type. But *TP53*, one tumor suppressor gene, were most frequently mutated across all stages in this cancer type. It seems the mutation of the oncogene alone cannot be tumorigenic, requiring the combination of the tumor suppressor gene to prompt cancer development. This was also observed in the “dominant‐waves” pattern. In BRCA, *PIK3CA* was an oncogene and dominated, while *TP53*,* GATA3*, and *MLL3* were waves and all of them were tumor suppressor genes. In the case of PAAD, *KRAS*, an oncogene, dominated and the tumor suppressor gene *TP53* showed two waves. In the “waves” pattern CESC, the first wave genes *EP300*,* MLL2*, and *MLL3* were all tumor suppressor genes, and the second wave genes *FBXW7* and *ATRX* were also tumor suppressor genes. In LIHC, also in “waves” pattern, the tumor suppressor gene *TP53* showed one wave, and the oncogene *CTNNB1* together with another event like the tumor suppressor gene *ARID1A* showed as another wave. In general, the observation in these nine cancer types indicated the mutation of the tumor suppressor genes appeared more like tumorigenic, and the mutation of the oncogenes seems requiring the combination of the tumor suppressor genes.

In summary, we revealed the genetic alterations of 138 driver genes in nine cancer types and determined three dynamic mutation changes patterns across different clinical stages, highlighting the hotspot regions on the coding sequences in the tumorigenesis. The potential clinical utility of this study is apparent.

## Conflict of Interest

The author declare that they have no conflict of interest.

## Supporting information


**Figure S1**. Correlation of nonsynonymous mutations with the disease stage in nine cancer types. Distribution of nonsynonymous mutations according to the disease stage of the patients in nine cancer types. *P* value was calculated by two‐sided Student's *t* test (mean ± SD).

**Figure S2**. Dynamic changes of the 140 random genes in different stages. We randomly selected 140 mutated genes in each cancer type and calculated the proportion of mutated patients harboring the corresponding genes in each clinical stage. The heat map showed that those genes showed dynamic changes across different stages.

**Figure S3**. Another four representative examples for the “dominant” pattern. In each cancer type, we calculated the proportion of mutated patients harboring the corresponding driver genes in each clinical stage. We labeled the mostly occurred genes across the entire stages (*TP53* in BLCA, *VHL* and *PBRM1* in KIRC, *KRAS* and *TP53* in LUAD, and *TP53* in STAD) and the mutated genes with relative lower proportion (*BAP1* and *SETD2* in KIRC, *ATM* and *STK11* in LUAD).

**Figure S4**. Temporal order relationship for the four cancer types. Based on the mutation spectrum for those mutated driver genes, we constructed the temporal order relationship using BML^1^ in each cancer type. Beginning with the normal circle, figure shows all possible evolutionary paths for driver genes after leaving out low‐probability (the probability from the parent circle to the child circle below 0.4) events. The arrow pointing means possible order relationship among mutated genes. Color for each circle is scaled according to the relative probability (decreasing from darker shade to light) from the parent circle to the child circle. The temporal order showed that *TP53* was the most initial mutated genes in BLCA and STAD which triggered all other genes, *VHL* dominated in KIRC, and *KARS* in LUAD.

**Figure S5**. Another representative example for the “dominant‐waves” and “waves” patterns. (A) Another representative example of the “dominant‐waves” pattern in PAAD. We calculated the proportion of mutated patients harboring the corresponding driver genes in each clinical stage (A) and the temporal order relationship (C). The mutations on *KRAS* dominated across all the stages, while *TP53* followed one wave of mutation and triggered the *SMAD4* at Stage IV. (B) Another representative example of the “waves” pattern in LIHC. We calculated the proportion of mutated patients harboring the corresponding driver genes in each clinical stage (B) and the temporal order relationship (D). The mutations on *TP53* served as one wave of mutation and *CTNNB1* followed as one another wave.

**Figure S6**. Distribution of codon changes of *TP53* in other five cancer types. We calculated the number of patients harboring each amino acids changes of *TP53* in each stage subgroup and checked the dynamic changes across clinical stages. We labeled the mutations occurred on the hotspot regions of the coding sequence of *TP53*.

**Figure S7**. Distribution of codon changes of *PIK3CA*. We calculated the number of patients harboring each amino acids changes of *PIK3CA* in each stage subgroup and checked the dynamic changes across clinical stages. We labeled the mutations occurred on the hotspot regions of the coding sequence of *PIK3CA*.

**Table S1**. List of the 138 driver genes.
