# Supplementary figures and images for: Dynamic changes of driver genes’ mutations across clinical stages in nine cancer types
Source: Cancer Med. 2016 Mar 19;5(7):1556–65. doi: 10.1002/cam4.704 (PMC4944883; doi:10.1002/cam4.704)

BLCA

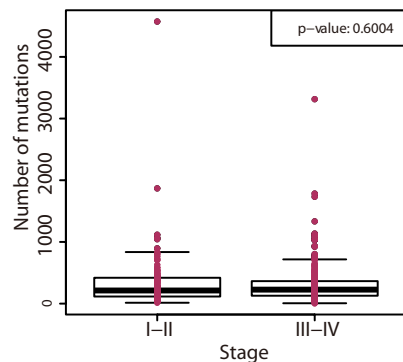

BRCA

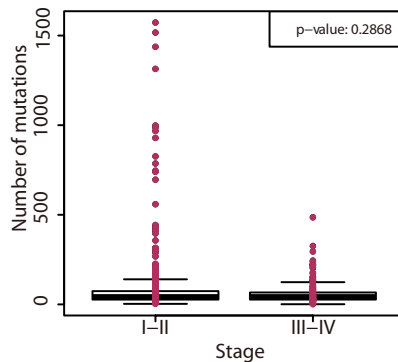

CESC

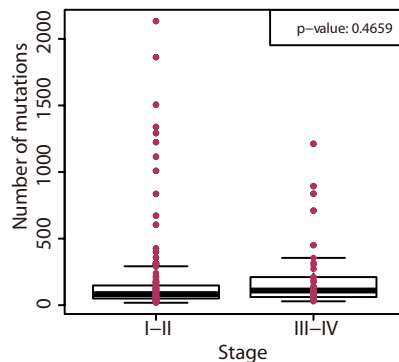

ESCA

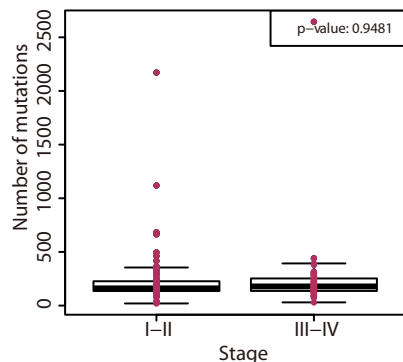

KIRC

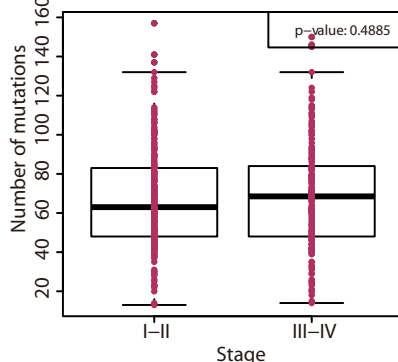

LIHC

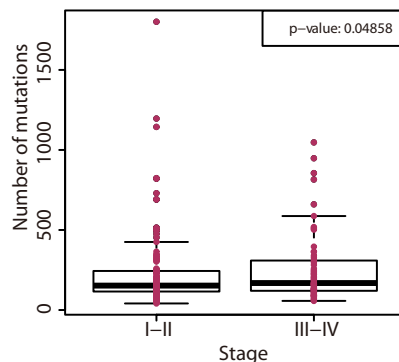

LUAD

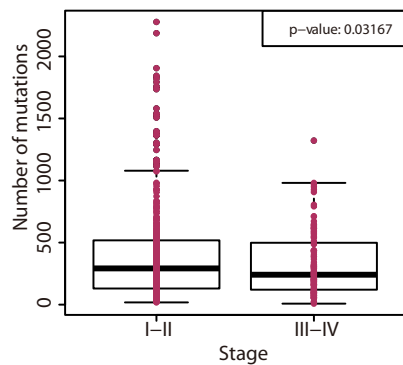

PAAD

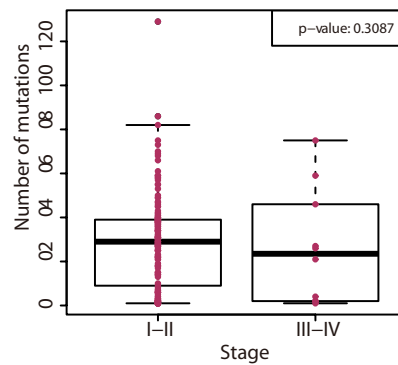

STAD

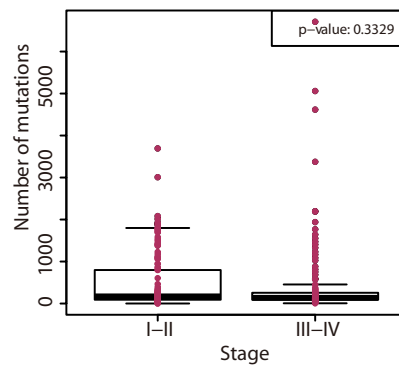

Supplement: Supplementary file 1 — Figure S1. Correlation of nonsynonymous mutations with the disease stage in nine cancer types. Distribution of nonsynonymous mutations according to the disease stage of the patients in nine cancer types. P value was calculated by two‐sided Student's t test (mean ± SD). [file CAM4-5-1556-s001.pdf]

BLCA

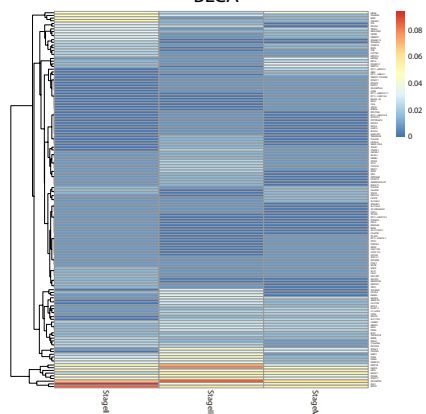

BRCA

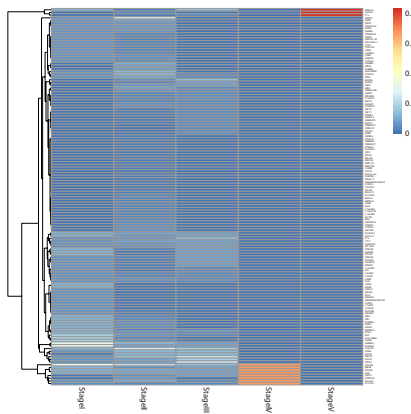

CESC

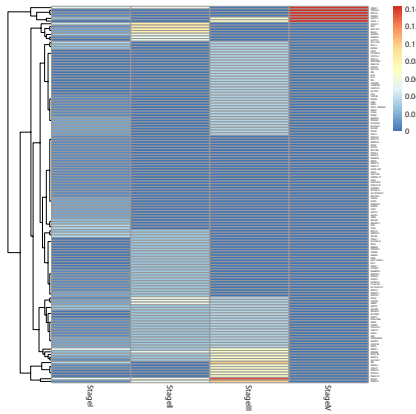

ESCA

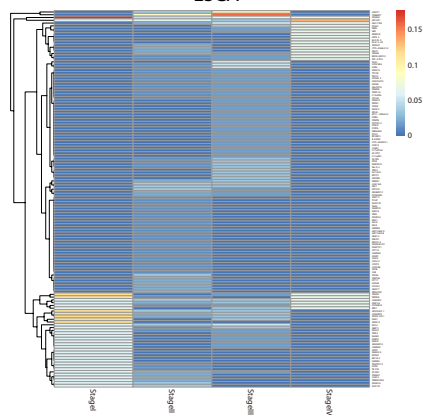

KIRC

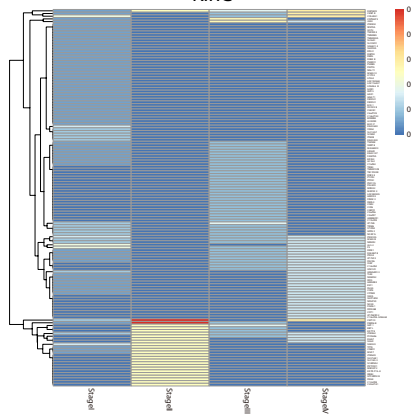

LIHC

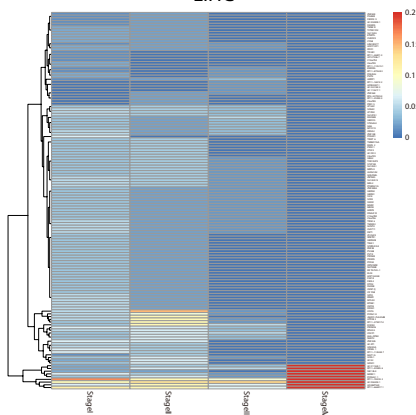

LUAD

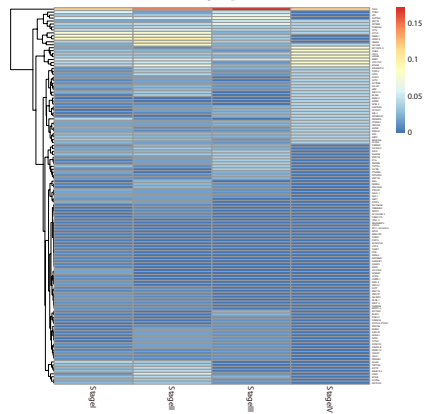

PAAD

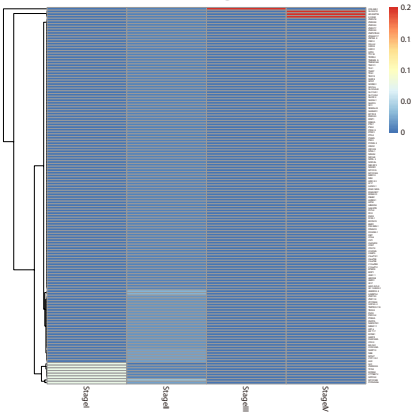

STAD

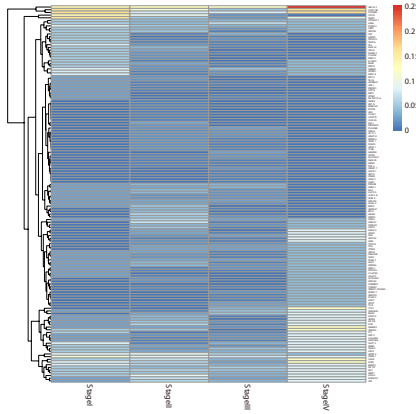

Supplement: Supplementary file 2 — Figure S2. Dynamic changes of the 140 random genes in different stages. We randomly selected 140 mutated genes in each cancer type and calculated the proportion of mutated patients harboring the corresponding genes in each clinical stage. The heat map showed that those genes showed dynamic changes across different stages. [file CAM4-5-1556-s002.pdf]

BLCA

TP53

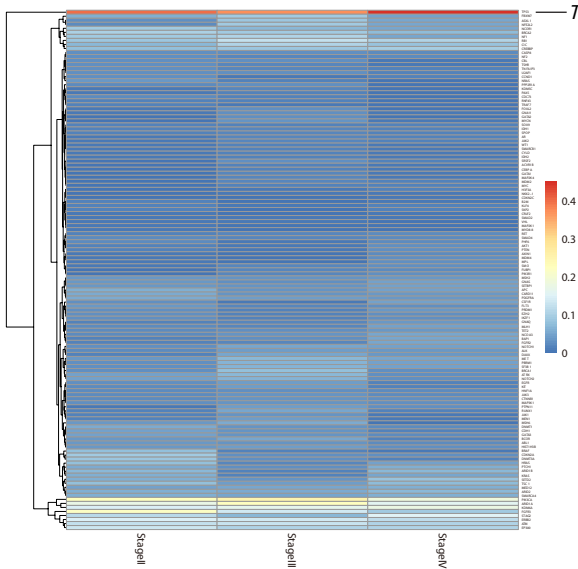

KIRC

BAP1  
SETD2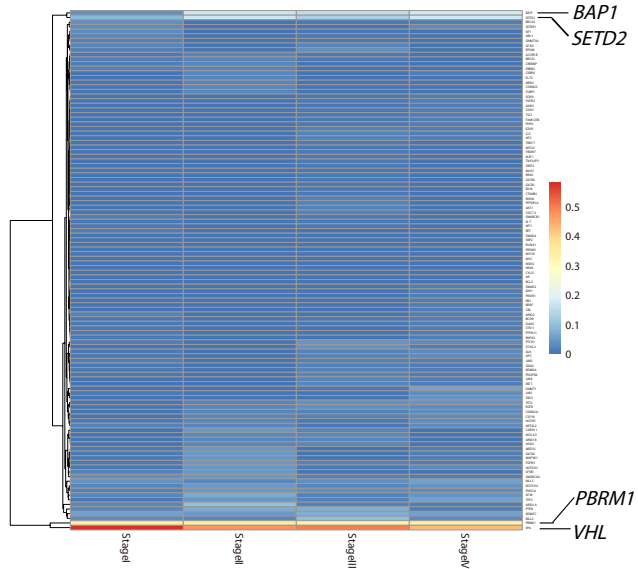

LUAD

ATM  
STK11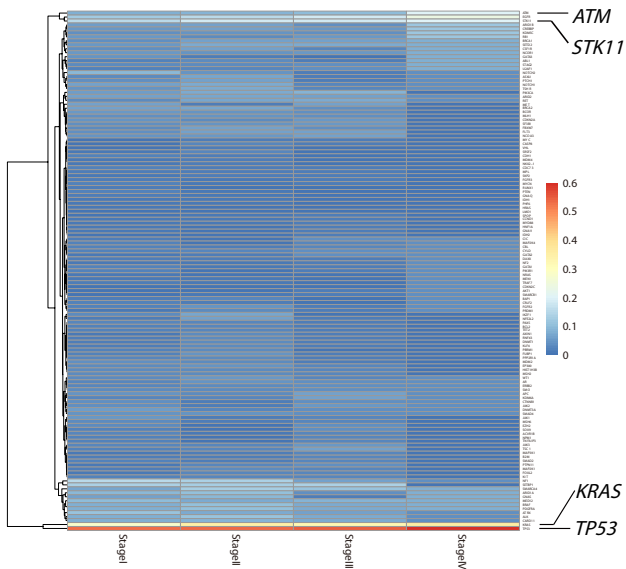

STAD

TP53

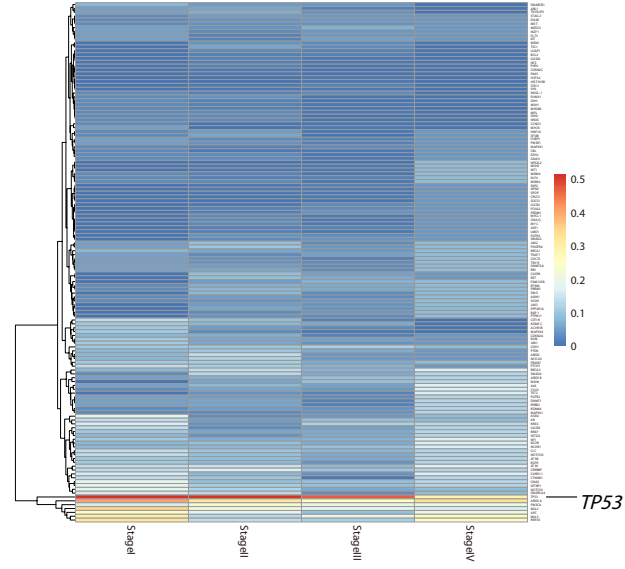

Supplement: Supplementary file 3 — Figure S3. Another four representative examples for the “dominant” pattern. In each cancer type, we calculated the proportion of mutated patients harboring the corresponding driver genes in each clinical stage. We labeled the mostly occurred genes across the entire stages (TP53 in BLCA, VHL and PBRM1 in KIRC, KRAS and TP53 in LUAD, and TP53 in STAD) and the mutated genes with relative lower proportion (BAP1 and SETD2 in KIRC, ATM and STK11 in LUAD). [file CAM4-5-1556-s003.pdf]

BLCA

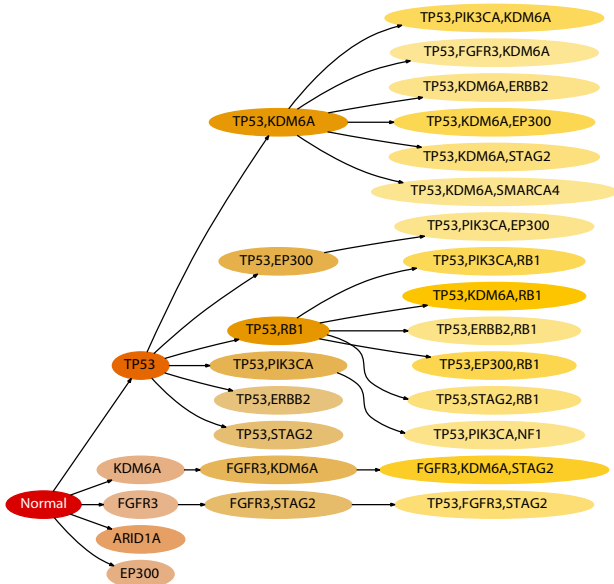

STAD

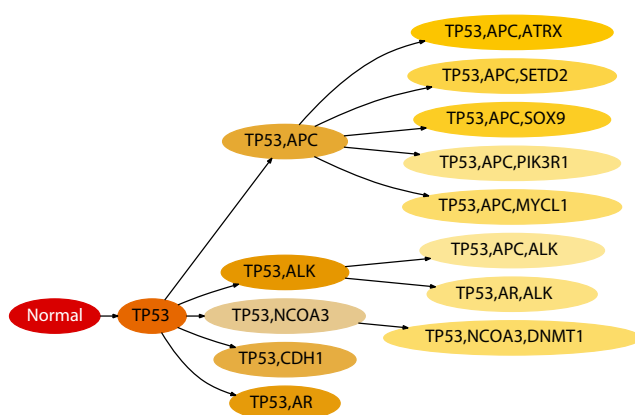

KIRC

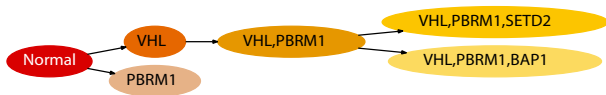

LUAD

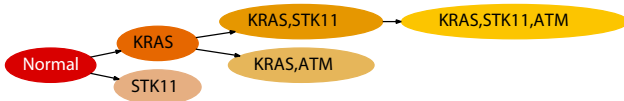

Supplement: Supplementary file 4 — Figure S4. Temporal order relationship for the four cancer types. Based on the mutation spectrum for those mutated driver genes, we constructed the temporal order relationship using BML1 in each cancer type. Beginning with the normal circle, figure shows all possible evolutionary paths for driver genes after leaving out low‐probability (the probability from the parent circle to the child circle below 0.4) events. The arrow pointing means possible order relationship among mutated genes. Color for each circle is scaled according to the relative probability (decreasing from darker shade to light) from the parent circle to the child circle. The temporal order showed that TP53 was the most initial mutated genes in BLCA and STAD which triggered all other genes, VHL dominated in KIRC, and KARS in LUAD. [file CAM4-5-1556-s004.pdf]

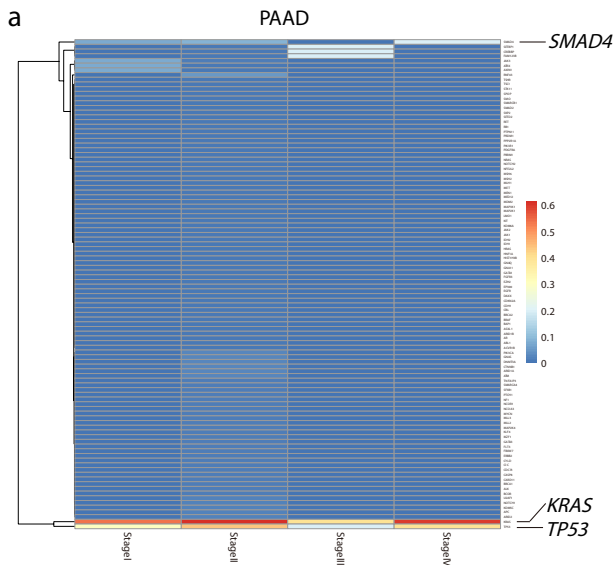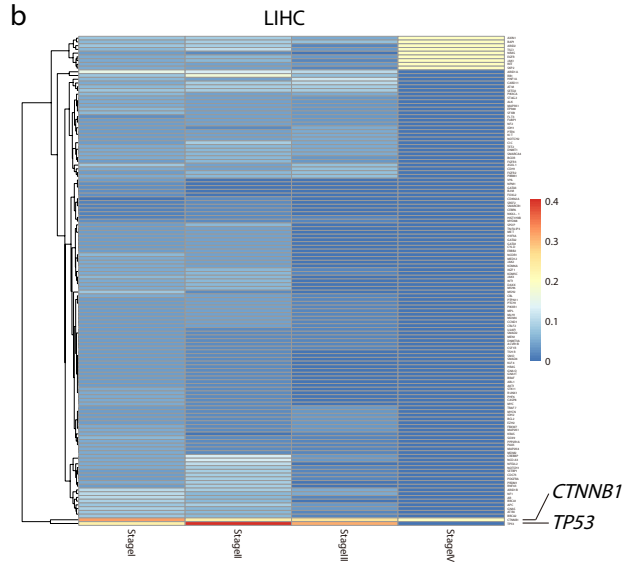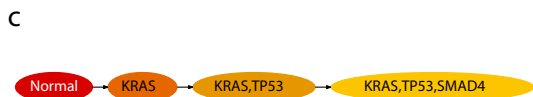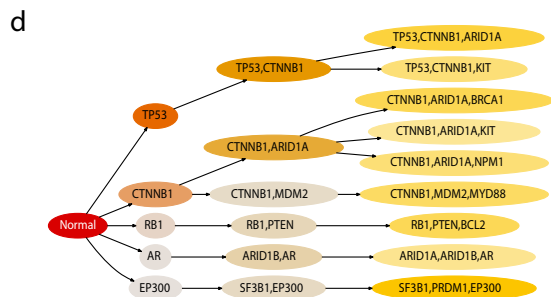

Supplement: Supplementary file 5 — Figure S5. Another representative example for the “dominant‐waves” and “waves” patterns. (A) Another representative example of the “dominant‐waves” pattern in PAAD. We calculated the proportion of mutated patients harboring the corresponding driver genes in each clinical stage (A) and the temporal order relationship (C). The mutations on KRAS dominated across all the stages, while TP53 followed one wave of mutation and triggered the SMAD4 at Stage IV. (B) Another representative example of the “waves” pattern in LIHC. We calculated the proportion of mutated patients harboring the corresponding driver genes in each clinical stage (B) and the temporal order relationship (D). The mutations on TP53 served as one wave of mutation and CTNNB1 followed as one another wave. [file CAM4-5-1556-s005.pdf]

ESCA

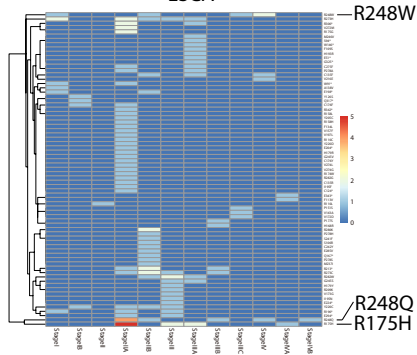

LIHC

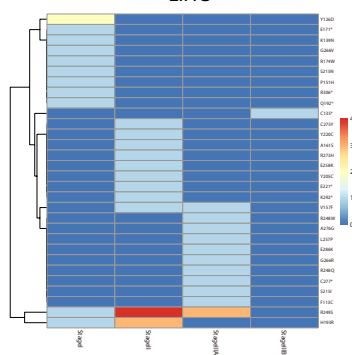

LUAD

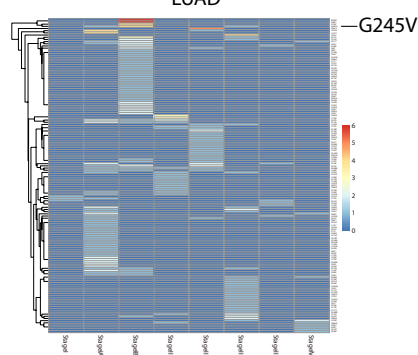

PAAD

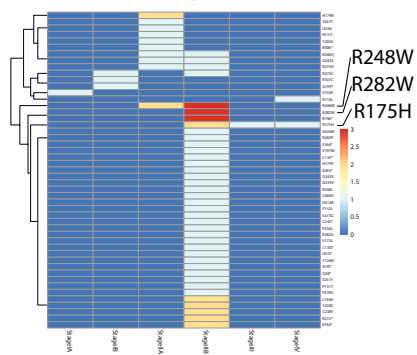

STAD

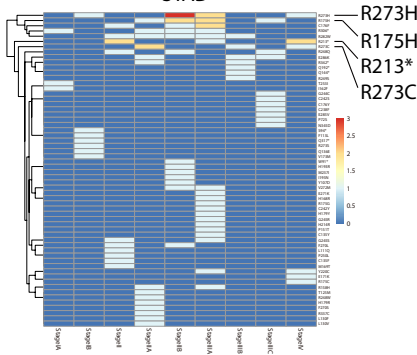

Supplement: Supplementary file 6 — Figure S6. Distribution of codon changes of TP53 in other five cancer types. We calculated the number of patients harboring each amino acids changes of TP53 in each stage subgroup and checked the dynamic changes across clinical stages. We labeled the mutations occurred on the hotspot regions of the coding sequence of TP53. [file CAM4-5-1556-s006.pdf]

BRCA

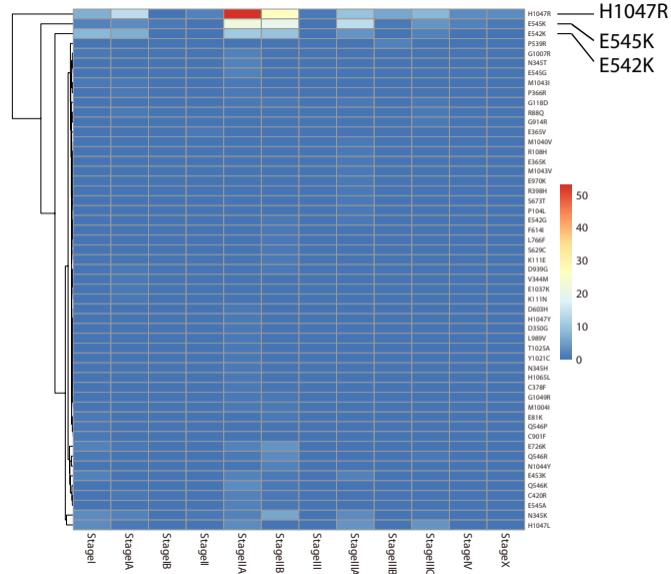

CESC

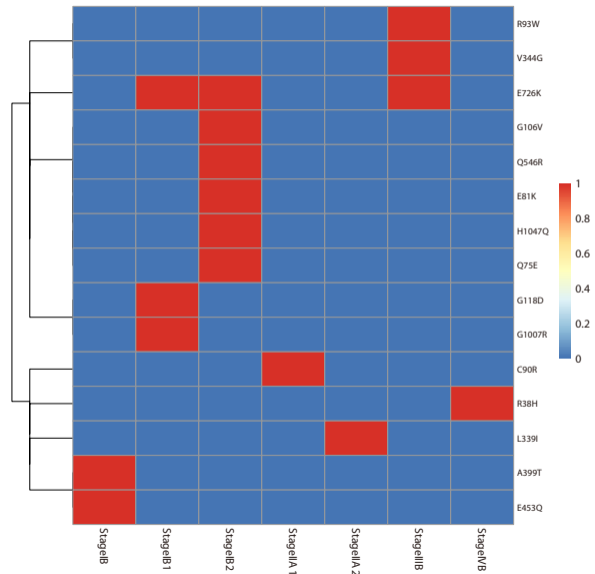

Supplement: Supplementary file 7 — Figure S7. Distribution of codon changes of PIK3CA. We calculated the number of patients harboring each amino acids changes of PIK3CA in each stage subgroup and checked the dynamic changes across clinical stages. We labeled the mutations occurred on the hotspot regions of the coding sequence of PIK3CA. [file CAM4-5-1556-s007.pdf]
