# Supplementary material for: Dynamic changes of driver genes’ mutations across clinical stages in nine cancer types
Source: Cancer Med. 2016 Mar 19;5(7):1556–65. doi: 10.1002/cam4.704 (PMC4944883; doi:10.1002/cam4.704)
Supplement: Supplementary file 8 — Table S1. List of the 138 driver genes. [file CAM4-5-1556-s008.doc]

Table S1. List of the 138 driver genes

| Gene Symbol^a^ | Gene Name^a^ | Classification^a^ | Cancer Type^b^ |
| --- | --- | --- | --- |
| *ABL1* | c-abl oncogene 1, receptor tyrosine kinase | Oncogene | chronic myoleid leukemia |
| *ACVR1B* | activin A receptor, type IB | Tumor suppressor gene | - |
| *AKT1* | v-akt murine thymoma viral oncogene homolog 1 | Oncogene | breast cancer |
| *ALK* | anaplastic lymphoma receptor tyrosine kinase | Oncogene | non-small cell lung cancer |
| *APC* | adenomatous polyposis coli | Tumor suppressor gene | colorectal adenocarcinoma |
| *AR* | androgen receptor | Oncogene | prostate cancer |
| *ARID1A* | AT rich interactive domain 1A (SWI-like) | Tumor suppressor gene | childhood cancer neuroblastoma |
| *ARID1B* | AT rich interactive domain 1B (SWI1-like) | Tumor suppressor gene | childhood cancer neuroblastoma |
| *ARID2* | AT rich interactive domain 2 (ARID, RFX-like) | Tumor suppressor gene | liver carcinoma |
| *ASXL1* | additional sex combs like 1 (Drosophila) | Tumor suppressor gene | hematological malignancies |
| *ATM* | similar to Serine-protein kinase ATM (Ataxia telangiectasia mutated) (A-T, mutated); ataxia telangiectasia mutated | Tumor suppressor gene | lung adenocarcinoma |
| *ATRX* | alpha thalassemia/mental retardation syndrome X-linked (RAD54 homolog, S. cerevisiae) | Tumor suppressor gene | lower-grade gliomas |
| *AXIN1* | axin 1 | Tumor suppressor gene | liver carcinoma |
| *B2M* | beta-2-microglobulin | Tumor suppressor gene | - |
| *BAP1* | BRCA1 associated protein-1 (ubiquitin carboxy-terminal hydrolase) | Tumor suppressor gene | renal cell carcinoma |
| *BCL2* | B-cell CLL/lymphoma 2 | Oncogene | breast cancer |
| *BCOR* | BCL6 co-repressor | Tumor suppressor gene | myelodysplastic syndromes |
| *BRAF* | v-raf murine sarcoma viral oncogene homolog B1 | Oncogene | colon cancer |
| *BRCA1* | breast cancer 1, early onset | Tumor suppressor gene | breast cancer |
| *BRCA2* | breast cancer 2, early onset | Tumor suppressor gene | breast cancer |
| *CARD11* | caspase recruitment domain family, member 11 | Oncogene | cutaneous squamous cell carcinoma |
| *CASP8* | caspase 8, apoptosis-related cysteine peptidase | Tumor suppressor gene | breast cancer |
| *CBL* | Cas-Br-M (murine) ecotropic retroviral transforming sequence | Oncogene | myeloproliferative disorders |
| *CDC73* | cell division cycle 73, Paf1/RNA polymerase II complex component, homolog (S. cerevisiae) | Tumor suppressor gene | hyperparathyroidism-Jaw tumor syndrome |
| *CDH1* | cadherin 1, type 1, E-cadherin (epithelial) | Tumor suppressor gene | breast cancer |
| *CDKN2A* | cyclin-dependent kinase inhibitor 2A (melanoma, p16, inhibits CDK4) | Tumor suppressor gene | lung cancer |
| *CEBPA* | CCAAT/enhancer binding protein (C/EBP), alpha | Tumor suppressor gene | acute myeloid leukemia |
| *CIC* | capicua homolog (Drosophila) | Tumor suppressor gene | brain cancer |
| *CREBBP* | CREB binding protein | Tumor suppressor gene | small-cell lung cancer |
| *CRLF2* | cytokine receptor-like factor 2 | Oncogene | - |
| *CSF1R* | colony stimulating factor 1 receptor | Oncogene | - |
| *CTNNB1* | catenin (cadherin-associated protein), beta 1, 88kDa | Oncogene | endometrioid endometrial carcinoma |
| *CYLD* | cylindromatosis (turban tumor syndrome) | Tumor suppressor gene | - |
| *DAXX* | death-domain associated protein | Tumor suppressor gene | pancreatic tumor |
| *DNMT1* | DNA (cytosine-5-)-methyltransferase 1 | Oncogene | - |
| *DNMT3A* | DNA (cytosine-5-)-methyltransferase 3 alpha | Oncogene | acute myeloid leukemia |
| *EGFR* | epidermal growth factor receptor (erythroblastic leukemia viral (v-erb-b) oncogene homolog, avian) | Oncogene | lung cancer |
| *EP300* | E1A binding protein p300 | Tumor suppressor gene | small-cell lung cancer |
| *ERBB2* | v-erb-b2 erythroblastic leukemia viral oncogene homolog 2, neuro/glioblastoma derived oncogene homolog (avian) | Oncogene | non-small-cell lung cancer |
| *EZH2* | enhancer of zeste homolog 2 (Drosophila) | Oncogene | prostate cancer |
| *FAM123B* | family with sequence similarity 123B | Tumor suppressor gene | colon and rectal cancer |
| *FBXW7* | F-box and WD repeat domain containing 7 | Tumor suppressor gene | melanoma |
| *FGFR2* | fibroblast growth factor receptor 2 | Oncogene | gastric cancer |
| *FGFR3* | fibroblast growth factor receptor 3 | Oncogene | urothelial cell carcinoma |
| *FLT3* | fms-related tyrosine kinase 3 | Oncogene | acute myeloid leukemia |
| *FOXL2* | forkhead box L2 | Oncogene | granulosa cell tumor |
| *FUBP1* | far upstream element (FUSE) binding protein 1 | Tumor suppressor gene | chronic lymphocytic leukemia |
| *GATA1* | GATA binding protein 1 (globin transcription factor 1) | Tumor suppressor gene | acute megakaryoblastic leukemia |
| *GATA2* | GATA binding protein 2 | Oncogene | prostate cancer |
| *GATA3* | GATA binding protein 3 | Tumor suppressor gene | breast cancer |
| *GNA11* | guanine nucleotide binding protein (G protein), alpha 11 (Gq class) | Oncogene | uveal melanoma |
| *GNAQ* | guanine nucleotide binding protein (G protein), q polypeptide | Oncogene | uveal melanoma |
| *GNAS* | GNAS complex locus | Oncogene | breast cancer |
| *H3F3A* | H3 histone, family 3B (H3.3B); H3 histone, family 3A pseudogene; H3 histone, family 3A; similar to H3 histone, family 3B; similar to histone H3.3B | Oncogene | giant cell tumors of bone |
| *HIST1H3B* | histone cluster 1, H3j; histone cluster 1, H3i; histone cluster 1, H3h; histone cluster 1, H3g; histone cluster 1, H3f; histone cluster 1, H3e; histone cluster 1, H3d; histone cluster 1, H3c; histone cluster 1, H3b; histone cluster 1, H3a; histone cluster 1, H2ad; histone cluster 2, H3a; histone cluster 2, H3c; histone cluster 2, H3d | Oncogene | pediatric glioma |
| *HNF1A* | HNF1 homeobox A | Tumor suppressor gene | hepatocellular adenomas |
| *HRAS* | v-Ha-ras Harvey rat sarcoma viral oncogene homolog | Oncogene | lung cancer |
| *IDH1* | isocitrate dehydrogenase 1 (NADP+), soluble | Oncogene | acute megakaryoblastic leukemia |
| *IDH2* | isocitrate dehydrogenase 2 (NADP+), mitochondrial | Oncogene | acute megakaryoblastic leukemia |
| *JAK1* | Janus kinase 1 | Oncogene | T-cell acute lymphoblastic leukemia |
| *JAK2* | Janus kinase 2 | Oncogene | Primary myelofibrosis |
| *JAK3* | Janus kinase 3 | Oncogene | - |
| *KDM5C* | lysine (K)-specific demethylase 5C | Tumor suppressor gene | clear cell renal cell carcinoma |
| *KDM6A* | lysine (K)-specific demethylase 6A | Tumor suppressor gene | bladder cancer |
| *KIT* | similar to Mast/stem cell growth factor receptor precursor (SCFR) (Proto-oncogene tyrosine-protein kinase Kit) (c-kit) (CD117 antigen); v-kit Hardy-Zuckerman 4 feline sarcoma viral oncogene homolog | Oncogene | - |
| *KLF4* | Kruppel-like factor 4 | Oncogene | B-cell lymphomas |
| *KRAS* | v-Ki-ras2 Kirsten rat sarcoma viral oncogene homolog | Oncogene | lung cancer |
| *MAP2K1* | mitogen-activated protein kinase kinase 1 | Oncogene | non-small-cell lung cancer |
| *MAP3K1* | mitogen-activated protein kinase kinase kinase 1 | Tumor suppressor gene | breast cancer |
| *MED12* | mediator complex subunit 12 | Oncogene | uterine leiomyomas |
| *MEN1* | multiple endocrine neoplasia I | Tumor suppressor gene | non-small-cell lung cancer |
| *MET* | met proto-oncogene (hepatocyte growth factor receptor) | Oncogene | non-small-cell lung cancer |
| *MLH1* | mutL homolog 1, colon cancer, nonpolyposis type 2 (E. coli) | Tumor suppressor gene | - |
| *MLL2* | myeloid/lymphoid or mixed-lineage leukemia 2 | Tumor suppressor gene | - |
| *MLL3* | myeloid/lymphoid or mixed-lineage leukemia 3 | Tumor suppressor gene | acute myeloid leukemia |
| *MPL* | myeloproliferative leukemia virus oncogene | Oncogene | primary myelofibrosis |
| *MSH2* | mutS homolog 2, colon cancer, nonpolyposis type 1 (E. coli) | Tumor suppressor gene | - |
| *MSH6* | mutS homolog 6 (E. coli) | Tumor suppressor gene | - |
| *MYD88* | myeloid differentiation primary response gene (88) | Oncogene | diffuse large B-cell lymphoma |
| *NCOR1* | nuclear receptor co-repressor 1 | Tumor suppressor gene | breast cancer |
| *NF1* | neurofibromin 1 | Tumor suppressor gene | breast cancer |
| *NF2* | neurofibromin 2 (merlin) | Tumor suppressor gene | Meningioma |
| *NFE2L2* | nuclear factor (erythroid-derived 2)-like 2 | Oncogene | liver carcinoma |
| *NOTCH1* | Notch homolog 1, translocation-associated (Drosophila) | Tumor suppressor gene | T-lymphoblastic leukemia |
| *NOTCH2* | Notch homolog 2 (Drosophila) | Tumor suppressor gene | chronic myelomonocytic leukemia |
| *NPM1* | nucleophosmin 1 (nucleolar phosphoprotein B23, numatrin) pseudogene 21; hypothetical LOC100131044; similar to nucleophosmin 1; nucleophosmin (nucleolar phosphoprotein B23, numatrin) | Tumor suppressor gene | acute myeloid leukaemia |
| *NRAS* | neuroblastoma RAS viral (v-ras) oncogene homolog | Oncogene | melanoma |
| *PAX5* | paired box 5 | Tumor suppressor gene | B-cell precursor acute lymphoblastic leukemia |
| *PBRM1* | polybromo 1 | Tumor suppressor gene | renal cell carcinoma |
| *PDGFRA* | platelet-derived growth factor receptor, alpha polypeptide | Oncogene | glioblastoma multiforme |
| *PHF6* | PHD finger protein 6 | Tumor suppressor gene | T-cell acute lymphoblastic leukemia |
| *PIK3CA* | phosphoinositide-3-kinase, catalytic, alpha polypeptide | Oncogene | breast cancer |
| *PIK3R1* | phosphoinositide-3-kinase, regulatory subunit 1 (alpha) | Tumor suppressor gene | breast cancer |
| *PPP2R1A* | protein phosphatase 2 (formerly 2A), regulatory subunit A, alpha isoform | Oncogene | ovarian and uterine carcinomas |
| *PRDM1* | PR domain containing 1, with ZNF domain | Tumor suppressor gene | B-cell lymphoma |
| *PTCH1* | patched homolog 1 (Drosophila) | Tumor suppressor gene | colorectal cancer |
| *PTEN* | phosphatase and tensin homolog; phosphatase and tensin homolog pseudogene 1 | Tumor suppressor gene | breast cancer |
| *PTPN11* | protein tyrosine phosphatase, non-receptor type 11; similar to protein tyrosine phosphatase, non-receptor type 11 | Oncogene | - |
| *RB1* | retinoblastoma 1 | Tumor suppressor gene | breast cancer |
| *RET* | ret proto-oncogene | Oncogene | lung adenocarcinoma |
| *RNF43* | ring finger protein 43 | Tumor suppressor gene | gastric cancer |
| *RUNX1* | runt-related transcription factor 1 | Tumor suppressor gene | neurofibromas |
| *SETD2* | SET domain containing 2 | Tumor suppressor gene | leukemia |
| *SETBP1* | SET binding protein 1 | Oncogene | myelodysplastic syndrome |
| *SF3B1* | splicing factor 3b, subunit 1, 155kDa | Oncogene | chronic lymphocytic leukemia |
| *SMAD2* | SMAD family member 2 | Tumor suppressor gene | - |
| *SMAD4* | SMAD family member 4 | Tumor suppressor gene | pancreatic ductal adenocarcinoma |
| *SMARCA4* | SWI/SNF related, matrix associated, actin dependent regulator of chromatin, subfamily a, member 4 | Tumor suppressor gene | non-small cell lung cancer |
| *SMARCB1* | SWI/SNF related, matrix associated, actin dependent regulator of chromatin, subfamily b, member 1 | Tumor suppressor gene | - |
| *SMO* | smoothened homolog (Drosophila) | Oncogene | - |
| *SOCS1* | suppressor of cytokine signaling 1 | Tumor suppressor gene | classical Hodgkin lymphoma |
| *SOX9* | SRY (sex determining region Y)-box 9 | Tumor suppressor gene | - |
| *SPOP* | speckle-type POZ protein | Oncogene | prostate cancer |
| *SRSF2* | SRSF2 serine/arginine-rich splicing factor 2 | Oncogene | - |
| *STAG2* | stromal antigen 2 | Tumor suppressor gene | pancreatic ductal adenocarcinoma |
| *STK11* | serine/threonine kinase 11 | Tumor suppressor gene | non-small cell lung cancer |
| *TET2* | tet oncogene family member 2 | Tumor suppressor gene | acute myeloid leukaemia |
| *TNFAIP3* | tumor necrosis factor, alpha-induced protein 3 | Tumor suppressor gene | - |
| *TRAF7* | TNF receptor-associated factor 7 | Tumor suppressor gene | meningiomas |
| *TP53* | tumor protein p53 | Tumor suppressor gene | breast cancer |
| *TSC1* | tuberous sclerosis 1 | Tumor suppressor gene | liver cancer |
| *TSHR* | thyroid stimulating hormone receptor | Oncogene | - |
| *U2AF1* | U2 small nuclear RNA auxiliary factor 1 | Oncogene | - |
| *VHL* | von Hippel-Lindau tumor suppressor | Tumor suppressor gene | renal cell carcinoma |
| *WT1* | Wilms tumor 1 | Tumor suppressor gene | Wilms tumor;aniridi;, genitourinary abnormalities; mental retardation |
| *CCND1* | cyclin D1 | Oncogene | liver carcinoma |
| *CDKN2C* | cyclin-dependent kinase inhibitor 2C (p18, inhibits CDK4) | Tumor suppressor gene | - |
| *IKZF1* | IKAROS family zinc finger 1 (Ikaros) | Tumor suppressor gene | - |
| *LMO1* | LIM domain only 1 (rhombotin 1) | Oncogene | T-Cell acute lymphoblastic leukemia |
| *MAP2K4* | mitogen-activated protein kinase kinase 4 | Tumor suppressor gene | prostate cancer |
| *MDM2* | Mdm2 p53 binding protein homolog (mouse) | Oncogene | - |
| *MDM4* | Mdm4 p53 binding protein homolog (mouse) | Oncogene | - |
| *MYC* | v-myc myelocytomatosis viral oncogene homolog (avian) | Oncogene | prostate cancer |
| *MYCL1* | v-myc myelocytomatosis viral oncogene homolog 1, lung carcinoma derived (avian) | Oncogene | small cell lung Cancer. |
| *MYCN* | v-myc myelocytomatosis viral related oncogene, neuroblastoma derived (avian) | Oncogene | neuroblastoma |
| *NCOA3* | nuclear receptor coactivator 3 | Oncogene | - |
| *NKX2-1* | NK2 homeobox 1 | Oncogene | - |
| *SKP2* | S-phase kinase-associated protein 2 (p45) | Oncogene | - |

^a^The 138 driver genes and the classfication of oncogene or tumor suppressor gene were obtained from previous report[^1^](#_ENREF_1).

^b^We scanned the literatures for these 138 genes and listed one example of reported cancer type in which the corresponding gene served as driver gene role.

**Supplementary References**

1. Vogelstein B, Papadopoulos N, Velculescu VE, Zhou S, Diaz LA, Jr., Kinzler KW. Cancer genome landscapes*. Science*. Mar 29 2013;339(6127):1546-1558.
