# Supplementary material for: Dynamic changes of driver genes’ mutations across clinical stages in nine cancer types
Source: Cancer Med. 2016 Mar 19;5(7):1556–65. doi: 10.1002/cam4.704 (PMC4944883; doi:10.1002/cam4.704)
Supplement: Supplementary file 9 [file CAM4-5-1556-s009.doc]

**Supplementary Figure Legends**

Supplementary Figure S1. Correlation of nonsynonymous mutations with the disease stage in nine cancer types. Distribution of nonsynonymous mutations according to the disease stage of the patients in nine cancer types. *P* value was calculated by two-sided Student’s *t* test (mean ± s.d.).

Supplementary Figure S2. Dynamic changes of the 140 random genes in different stages. We randomly selected 140 mutated genes in each cancer type and calculated the proportion of mutated patients harboring the corresponding genes in each clinical stage. The heatmap showed that those genes showed dynamic changes across different stages.

Supplementary Figure S3. Another four representative examples for the “dominant” pattern. In each cancer type, we calculated the proportion of mutated patients harboring the corresponding driver genes in each clinical stage. We labeled the mostly occurred genes across the entire stages (*TP53* in BLCA, *VHL* and *PBRM1* in KIRC, *KRAS* and *TP53* in LUAD, and *TP53* in STAD) and the mutated genes with relative lower proportion (*BAP1* and *SETD2* in KIRC, *ATM* and *STK11* in LUAD).

Supplementary Figure S4. Temporal order relationships for the four cancer types. Based on the mutation spectrum for those mutated driver genes, we constructed the temporal order relationship using BML[^1^](#_ENREF_1) in each cancer type. Beginning with the normal circle, figure shows all possible evolutionary path for driver genes after leaving out low-probability (the probability from the parent circle to the child circle below 0.4) events. The arrow pointing means possible order relationship among mutated genes. Color for each circle is scaled according to the relative probability (decreasing from darker shade to light) from the parent circle to the child circle. The temporal order showed that *TP53* was the most initial mutated genes in BLCA and STAD which triggering all other genes, *VHL* dominated in KIRC and *KARS* in LUAD.

Supplementary Figure S5. Another representative example for the “dominant-waves” and “waves” patterns. (a) Another representative example of the “dominant-waves” pattern in PAAD. We calculated the proportion of mutated patients harboring the corresponding driver genes in each clinical stage (a) and the temporal order relationship (c). The mutations on *KRAS* dominated across all the stages while *TP53* followed one wave of mutation and triggered the *SMAD4* at Stage IV. (b) Another representative example of the “waves” pattern in LIHC. We calculated the proportion of mutated patients harboring the corresponding driver genes in each clinical stage (b) and the temporal order relationship (d). The mutations on *TP53* served as one wave of mutation and *CTNNB1* followed as one another wave.

Supplementary Figure S6. Distribution of codon changes of *TP53* in other five cancer types. We calculated the number of patients harboring each amino acids changes of *TP53* in each stage subgroup and checked the dynamic changes across clinical stages. We labeled the mutations occurred on the hot-spot regions of the coding sequence of *TP53.*

Supplementary Figure S7. Distribution of codon changes of *PIK3CA*. We calculated the number of patients harboring each amino acids changes of *PIK3CA* in each stage subgroup and checked the dynamic changes across clinical stages. We labeled the mutations occurred on the hot-spot regions of the coding sequence of *PIK3CA.*

**Supplementary References**

1. Misra N, Szczurek E, Vingron M. Inferring the paths of somatic evolution in cancer. *Bioinformatics.* Sep 1 2014;30(17):2456-2463.
